# A multicenter prospective observational multi-omics study protocol to identify biomarkers for severe dengue: COMBAT study clinical protocol

**DOI:** 10.1186/s12879-026-14111-x

**Published:** 2026-07-31

**Authors:** Piya Paul Mudgal, Silvia Patricia Zuniga Veliz, Magda Lourda, Trupti Satish Kadni, Muralidhar Varma, Anup Mazumder, Sandeep Budhiraja, Namita Jaggi, Chiranjay Mukhopadhyay, Sagar Sengupta, Arindam Maitra, Kristina Hug, Indranil Sinha, Anoop T. Ambikan, Iva Filipovic, Ujjwal Neogi

**Affiliations:** 1 Manipal Institute of Virology, Manipal Academy of Higher Education, Manipal, India; 2 BioBox Guatemala, Guatemala City, Guatemala; 3 Center for Systems Infection Biology, Department of Laboratory Medicine, Karolinska Institutet, Stockholm, Sweden; 4 Department of Infectious Diseases, Kasturba Medical College, Manipal, India; 5 Biotechnology Research and Innovation Council - National Institute of Biomedical Genomics (BRIC-NIBMG), Kalyani, West Bengal 741251 India; 6 Max Super Speciality Hospital (A Unit of Devki Devi Foundation), Max Healthcare, Delhi, 110017 India; 7 Infection Control, Artemis Hospital, Delhi, India; 8 Medical Ethics, Department of Clinical Sciences, Lund Faculty of Medicine, Lund University, BMC I12, Lund, 22184 Sweden; 9 Division of Clinical microbiology, Department of Laboratory Medicine, Karolinska Institutet, Stockholm, Sweden; 10 Division of Molecular Metabolism, Department of Medical Biochemistry & Biophysics , Karolinska Institutet Karolinska Institutet, Stockholm, Sweden; 11 Amsterdam University Medical Center, Location Academic Medical Center, University of Amsterdam, Amsterdam, The Netherlands; 12 University of Oslo, Oslo, Norway; 13 Justus Liebig University Giessen, Giessen, Germany; 14 React4Life, Genova, Italy; 15 Olink Ab, Uppsala, Sweden; 16 ChipNano Imaging Ab, Tromsø, Norway; 17 Genos, Zagreb, Croatia; 18 University of Missouri, Columbia, USA; 19 Regional Center for Biotechnology, Faridabad, India

**Keywords:** Dengue, Severe dengue, Biomarkers, Transcriptomics, Proteomics, Glycomics, Metabolomics, Multi-omics, Systems biology, Public health surveillance

## Abstract

**Background:**

Dengue virus (DENV) infection poses a major global public health burden, particularly in endemic regions where repeated exposure increases the risk of severe disease. Despite revisions to the World Health Organization (WHO) dengue classification, early prediction of progression to severe dengue remains challenging due to overlapping clinical and laboratory features. Current management strategies rely primarily on supportive care and reactive monitoring, underscoring the need for predictive biomarkers that enable early risk stratification and timely intervention.

**Methods:**

COMBAT is a prospective, multicenter, observational longitudinal study conducted in dengue-endemic regions of Guatemala and India. Patients will be classified according to WHO 2009 criteria into dengue without warning signs, dengue with warning signs, and severe dengue, alongside age- and sex-matched healthy controls. A single blood sample will be collected from non-hospitalized patients while two blood samples per participant will be collected during hospitalization and one at discharge. Multi-omics analyses, including transcriptomics, proteomics, glycomics and metabolomics, will be performed in a discovery cohort and validated in an independent cohort. Integrated systems biology approaches will be used to identify host immune and metabolic pathways associated with dengue severity in a mechanism-based prognostic biomarker discovery.

**Discussion:**

This study aims to generate comprehensive systems-level insights into host–virus interactions driving dengue severity and to identify biomarkers predictive of disease progression. The findings may inform improved patient triage, early intervention strategies, and the identification of novel therapeutic targets.

**Trial registration:**

ClinicalTrials.gov ID NCT06751836 Registration Date: December 13, 2024, CTRI/2022/10/046293 (MAHE) Registration Date: October 10, 2022.

## Background

Dengue is a mosquito-borne viral infection with a wide clinical spectrum ranging from self-limiting febrile illness to life-threatening severe dengue characterized by plasma leakage, hemorrhage, and organ failure [1]. Transmitted primarily by *Aedes aegypti* and *Aedes albopictus* mosquitoes, the virus encompasses four antigenically distinct serotypes (DENV-1 through DENV-4), each capable of inducing both primary infection and, upon subsequent exposure to a heterologous serotype, a more severe immunopathological response [2]. Globally, dengue continues to expand geographically, placing nearly half of the world’s population at risk. The World Health Organization (WHO) estimates approximately 390 million dengue infections annually, of which roughly 96 million manifests clinically (WHO fact sheet, Dengue August 2025, https://www.who.int/news-room/fact-sheets/detail/dengue-and-severe-dengue). Over the past two decades, reported case counts have increased eight-fold, driven by urbanization, increased international travel, climate change, and inadequate vector control measures. Dengue is now endemic in more than 100 countries across tropical and subtropical regions of the Americas, South-East Asia, the Western Pacific, Africa, and the Eastern Mediterranean.

Early identification of patients at risk of severe disease remains one of the most pressing challenges in dengue clinical management [3, 4]. The clinical course of dengue is characteristically triphasic, encompassing a febrile phase (days 1–3), a critical phase (days 4–6) during which plasma leakage may precipitate dengue shock syndrome, and a recovery phase. The overlap of clinical features across severity strata particularly during the early febrile phase renders timely risk stratification both critical and elusive.

Despite the 2009 WHO dengue classification system, which introduced “warning signs” as clinical predictors of severe dengue, evidence regarding their individual and combined predictive value remains inconsistent [3, 5]. Laboratory markers such as leukopenia, thrombocytopenia, and elevated hematocrit are routinely monitored; however, their sensitivity and specificity for predicting progression to severe dengue are suboptimal when assessed in isolation. Biomarkers including NS1 antigen levels, inflammatory cytokines, and endothelial permeability indices have shown promise in exploratory studies but have yet to be validated prospectively in diverse clinical settings [3, 6].

No validated, widely applicable clinical prediction tool currently exists that integrates bedside clinical features, routine laboratory parameters, and emerging biomarkers to accurately stratify patients by severity risk, despite progress in understanding dengue pathophysiology. This gap results in significant heterogeneity in clinical decision-making ranging from unnecessary hospitalizations that strain healthcare resources to premature discharge of high-risk patients [7]. The consequences are particularly severe in resource-limited endemic settings, where intensive care capacity is constrained and case fatality rates can exceed those in well-resourced environments [8]. 

Recent advances in high-throughput multi-omics technologies have fundamentally transformed the capacity to interrogate host responses to infectious diseases. The ability to simultaneously characterize transcriptomic, proteomic, and metabolomic alterations from a single biological sample offers an unprecedented systems-level perspective on host–pathogen interactions, enabling the identification of molecular signatures that transcend single-platform observations and more faithfully capture the biological complexity underlying disease severity [9]. 

The COMBAT study aims to address this gap by employing a multidisciplinary, systems biology approach to identify molecular signatures and pathways associated with dengue severity across geographically distinct endemic regions. The identification of predictive biomarkers and mechanistic pathways may support earlier dengue risk stratification, improve clinical management, and inform the development of novel therapeutic strategies.

## Methods/design

### Study aim

The COMBAT clinical study aims to identify and validate host molecular biomarkers and dysregulated biological pathways associated with dengue disease severity using an integrated multi-omics systems biology approach in dengue-endemic populations.

#### Primary objectives

1. To determine dengue virus serotypes and characterize host cytokine profiles across dengue severity categories.
2. To identify transcriptomic, proteomic, metabolomic, and glycomic signatures associated with dengue disease severity.

#### Secondary objectives

1. To integrate transcriptomic, proteomic, metabolomic, and glycomic data to identify dysregulated immune and metabolic pathways associated with disease progression.
2. To validate candidate biomarkers and pathways in geographically independent cohorts.
3. To investigate mechanistic host pathways influencing dengue severity using in vitro models informed by clinical multi-omics findings.

### Study design

COMBAT is a prospective, multicenter, observational longitudinal study designed to characterize host responses to dengue infection without altering standard clinical management.

### Study setting

The study is conducted at clinical sites in two dengue-endemic regions, Guatemala (Central America) and India (Southeast Asia). Guatemala is an exemplary location for studying the DENV infection, owing to its distinctive combination of climatic diversity and endemic and dengue presence. Samples are collected from different hospitals following report of the outbreak. Under the India-EU cooperation on research & innovation (R&I) and co-funding partnership under the EU framework program on R&I “Horizon Europe”, samples are also collected from three hospitals in India, i.e., the Artemis Hospital and Max Health Care (a unit of Devki Devi Foundation) in New Delhi and Kasturba Hospital, Manipal, Karnataka (with diagnostic facility at Manipal Institute of Virology, MAHE, Manipal, Karnataka). Those are the two epicentres of the dengue endemic in India.

### Study cohorts

Blood samples from patients are collected as per the WHO revised 2009 case classification [1] as: (1) dengue without warning signs (DwoWS), (2) dengue with warning signs (DWS) and (3) severe dengue (SD). Additionally, non-dengue samples will be collected from DENV-negative healthy controls (HCs). 100 individuals from each category will be collected in Guatemala and will serve as the discovery cohort, while 150 individuals from each category will be collected from India and serve as the validation cohort. The patient inclusion and exclusion criteria are presented in Table 1. Additional cohorts from Guatemala, Brazil and Honduras will be used for further validation of the dengue severity biomarkers. Validation of any additional cohorts requires a local ethical clearance, as well as approval from both the Swedish Ethical Review Authority and the COMBAT General Assembly.

**Table 1:** Inclusion and Exclusion Criteria for patient enrolment

Inclusion Criteria: The criteria for enrolling the patients in the respective groups.
**Group 1**: Dengue without warning signs (DwoWS) Patients presenting with the following symptoms and confirmed positive for dengue infection following anti-dengue IgM or NS1 ELISA (Enzyme-linked Immunosorbent Assay) lab tests. Symptoms are fever along with at least two of the following: • Myalgia, arthralgia • Nausea and/or vomiting • Rashes • Leukopenia
**Group 2**: DENV with warning signs (DWS) Patients with the above symptoms and any of the following clinical signs: • Abdominal pain with tenderness • Persistent vomiting • Fluid accumulation • Mucosal bleeding • Restlessness, lethargy • Liver enlargement >2 cm • Laboratory findings: An increase in hematocrit levels and a rapid decrease in platelet count
**Group 3**: Severe dengue (SD) Patients with: • Severe plasma leakage leading to hypovolemic shock (dengue shock syndrome) and fluid accumulation with respiratory distress • Severe bleeding, such as epistaxis, and internal bleeding, evaluated by a clinician • Severe organ involvement with any of the following symptoms: - Liver AST or ALT ≥ 1000 - CNS: impaired consciousness - Heart and other organs failure
**Exclusion criteria**: Individuals with the following characteristics were not enrolled.
• Patients below the age of 18 years • Pregnant, immunocompromised or undergoing chemotherapy, on steroids or immunosuppressive drugs in the last year or with liver cirrhosis • Co-infection with a non-dengue pathogen • Cases with a lab-confirmed dengue infection within the past three months

The study is designed to investigate the progression of dengue infection in patients, from without warning signs, to with warning signs to severe dengue, which varies between cases. If a patient recruited in the category “DwoWS” develops symptoms correlating with severity and receives intensive care, they will then be re-categorized into the second “DWS” or third “SD” group. Keeping in mind the complexity of dengue presentation, subcategorization of the patients will be performed as necessary to establish the degree of severity depending on the timepoint of recruitment and the points of sample collection. The highest (peak) severity category reached during the acute illness constitutes the participant’s definitive severity classification and serves as the primary outcome label for all biomarker analyses. For incorporation into the biomarker discovery and validation analyses, the earliest sample for each participant collected before or at the point of clinical deterioration is used as the predictor, and the definitive peak severity category is used as the outcome.

### Recruitment of controls

Healthy participants (*n* = 100 from Guatemala and *n* = 150 from India) who have not had a febrile episode in the last three months are enrolled as HCs to the cohort, which will further be age- and sex- matched. Samples from these participants are tested for anti-dengue IgM and NS1 ELISA to rule out ongoing asymptomatic dengue infection, and anti-dengue IgG ELISA to check for past dengue infection. Each of the control participants is also asked to provide a detailed medical history.

### Study procedures

A single blood sample is collected from the non-hospitalized patients, while two blood samples per participant are collected from the hospitalized patients. The first one is taken within the first two days of hospitalization, and the second at discharge. A questionnaire is administered at the time of blood collection (Table 2). Total participation time is less than 15 min. For Guatemala, whole blood is collected in Tempus™ tube for transcriptomics and in Cytodelics medium for flow cytometry, while plasma from EDTA blood and serum are collected for proteomics, metabolomics and glycomics. For India, whole blood (3 ml) is collected in Tempus™ tubes for transcriptomics and 7–8 ml in BD Vacutainer CPT™ Cell Preparation Tubes for plasma separation and PBMC isolation.

**Table 2:** Routine clinical investigations performed in dengue cases in all the cohorts

Parameters	Unit of Measurement
Age	In years
Sex	Male/Female
**Disease symptoms at the time of sampling**	
Duration of symptoms onset at sample collection	In days
Duration of hospitalization at sample collection	In days
Fever	Yes/No
Body pain	Yes/No
Joint pain (arthralgia)	Yes/No
Rash	Yes/No
Nausea/vomiting	Yes/No
Night sweats	Yes/No
Retro-orbital pain	Yes/No
Abdominal pain	Yes/No
Headache	Yes/No
Chills/ rigor	Yes/No
Burning micturition	Yes/No
Dizziness	Yes/No
**Diet/Lifestyle**	
Vegetarian	Yes/No
Meat eater	Yes/No
Alcohol	Yes/No
Smoking	Yes/No
**Clinical biochemistry**	
Hemoglobin	g/dL
White Blood Cells	(value)/µL
Differential Lymphocyte Count (eosinophils, neutrophils, lymphocytes)	(value)/ µL
Platelets	(value)/ µL
Hematocrit	%
Procalcitonin	mcg/L
Total bilirubin	mg/dL
Aspartate Transaminase	IU/L
Alanine Transaminase	IU/L
Alkaline Phosphatase	U/L
Urea	mg/dL
Creatinine	mg/dL
Partial Thromboplastin Time	seconds
Activated Partial Thromboplastin Time	seconds
Serum Lactate Dehydrogenase	U/L
Lactate Oxidase (Arterial Blood Gas)	mg/dL
Creatinine Phosphokinase	U/L
Troponin T	ng/mL
N-Terminal Pro- B-Type Natriuretic Protein	pg/mL

#### Multi-omics studies and biomarker identification

We will perform RNA sequencing (RNA-seq) of the blood cells [10], proteomics by Olink^®^ Reveal, and metabolomics using liquid chromatography-mass spectrometry (LC-MS/MS) in the samples collected from Guatemala [11, 12]. Due to different numbers of analytes, i.e., genes from RNA-seq (n = ~ 20000), protein (n = ~ 1034) and metabolites (n = ~ 1200), we will first identify biomarkers associated with disease severity in each omics dataset using Random Forest for feature selection, and Bayesian belief network (BBN) based structural causal modelling to infer putative causal relationships [12]. To further refine the biomarkers, we will perform integrative analysis using the Consensus Association (CA) analysis and gene/protein/metabolite cooperation networks by integrating the transcriptomics, proteomics and metabolomics analysis to identify a robust mechanism-informed protein biomarker panel for clinical translation [9]. Additionally, a high-throughput analysis of total plasma, IgG, and/or IgM N-glycome [13, 14] from the available patient cohorts will be performed to evaluate glycosylation as a biomarker of disease severity. For Indian samples we will perform RNA-seq of the blood cells and proteomics by Olink^®^ Reveal.

### Single cell RNA sequencing (scRNA-seq)

In a subset of samples (~ 20–25%) scRNA-seq will be performed using two different platforms to generate cross-validated data and to establish a scalable framework for future dengue infection studies as well as future pandemic preparedness. In the Guatemala cohort, instrument-free Parse Biosciences Evercode platform will be used for large-scale sample multiplexing and transcriptomic profiling through split-pool combinatorial barcoding, while minimizing batch effects and improving cohort-level comparability. In the Indian cohort, microfluidics-based 10x Genomics Chromium scRNA-seq protocol will be used to generate high-resolution transcriptomic profiles and resolve rare or functionally distinct cell populations. Running both in parallel will allow us to benchmark performance on matched infectious diseases questions, assess cross-platform results, and establish reproducible and scalable workflows that can be rapidly deployed in future outbreak responses. Following sequencing, reads will be processed using platform-specific pipelines, followed by harmonized quality control, normalization, dimensionality reduction, clustering, and cell-type annotation. Differential gene expression, pathway enrichment, trajectory analysis, regulatory network analysis and cell–cell communication analyses will be applied across both cohorts to identify disease-associated cellular states, regulatory programs, and intercellular signalling networks.

#### Development of the DENV specific genome-scale metabolic model (GEM)

**Fig. 1:**
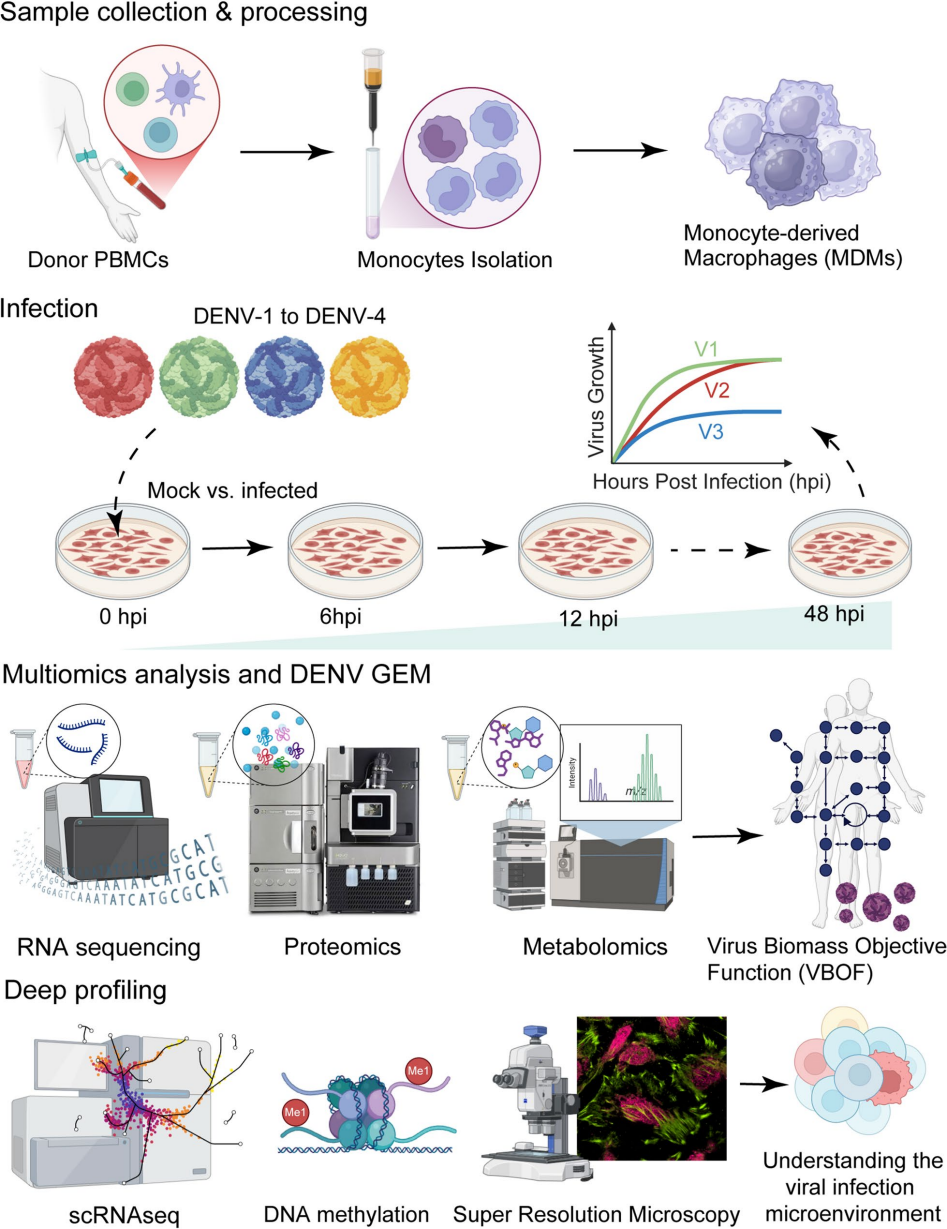
Ex vivo GEM and deep profiling of viral infection microenvironment

We will first develop the DENV specific GEM by infecting the human monocyte derived macrophages (MDMs) with all four dengue serotypes (DENV-1 to DENV-4). Anonymized PBMCs are also collected from the healthy blood donors for in vitro infection experiments. MDMs will be infected and samples will be collected at 6 h, 12 h, 24 h, 36 h and 48 h post-infection. Transcriptomics, proteomics and metabolomics will be performed to develop the DENV GEM as described by us previously in SARS-CoV-2 [15]. Additionally, we will perform scRNA-seq, assess DNA methylation changes by bisulfite pyrosequencing, and utilize super-resolution microscopy to characterize the microenvironment during viral infection (Fig. 1).

#### Personalized GEM to bridge and improve the ex-vivo cell-based DENV-GEM

To further improve a DENV GEM developed in an ex vivo assay, we will add the patient-based GEM both from bulk sequencing and scRNA-seq data to improve the predictability of the immunomodulator. We will use the personalized GEM developed recently to avoid heterogeneity by adding the viral biomass objective function (VBOF). We will provide feedback on the essential gene-metabolite knock-out (KO) module to the initial DENV-GEM to improve the identification of the immunomodulator [10].

#### Artificial intelligence (AI) models for DENV severity

The assessment of DENV severity using traditional methods has proven difficult due to many contributing factors, such as variation in symptoms, dynamic nature of dengue infection, and lack of specific biomarkers, amongst others [8]. Multiple machine-learning and deep-learning models, including logistic regression, random forests, XGBoost, support vector machines, multilayer perceptrons, and multimodal neural-network architectures, will be trained and systematically compared for DENV severity prediction. The models will integrate available clinical, laboratory, demographic, virological, and multi-omics data, using feature-selection and dimensionality-reduction approaches. Model interpretability will be assessed using explainable AI approaches, including SHapley Additive exPlanations (SHAP) and Local Interpretable Model-agnostic Explanations (LIME), to identify the variables and molecular features driving individual- and cohort-level predictions. Temporal and geographical robustness will be evaluated using time-split validation, external validation across sites, and leave-one-site-out cross-validation. Model performance will be assessed using AUROC, AUPRC, sensitivity, specificity, F1-score, calibration, and decision-curve analysis to identify clinically robust and generalizable models for DENV severity prediction.

#### Development of dengue severity Flex panel

As the main objective of the dengue severity panel is clinical deployment, we will develop a focused proteomics panel of 5–10 biomarkers derived from the mechanism-informed biomarker discovery that follows our integrative omics analysis of severe dengue. Discovery will be performed using the Olink^®^ Reveal platform, which profiles 1034 proteins, and the resulting candidates will be carried forward into a targeted Olink^®^ Flex assay containing the 5–10 prioritized biomarkers. The panel will be validated in both the Guatemala and India cohorts, with subsequent validation in additional independent cohorts.

### Statistical analysis

Differential expression analyses will be conducted separately for transcriptomic, proteomic, metabolomic and glycomic datasets with correction for multiple hypothesis testing. Integrated pathway and network analyses will be used to identify dysregulated biological processes associated with disease severity. Validation analyses will be performed using independent cohorts. As there are no high-dimensional studies with such large proteomics (> 1000 proteins) and metabolomics (> 1000 metabolites) in dengue, we have used the RNASeqSampleSize [16] to calculate the sample size for the RNA sequencing based on prior data for other viruses. Prior data indicates that the minimum average read counts among the prognostic genes in the control group is 5, the maximum dispersion is 0.5 and the ratio of the geometric mean of normalization factors is 1. If the total number of genes for testing is 20,000, the top 100 genes are prognostic and the desired minimum fold change is 2, then we will need to study 67 subjects in each group to reject the null hypothesis that the population means of the two groups are equal with probability (power) 0.8 using the Fisher’s exact test. The FDR associated with this test of this null hypothesis is 0.01. Assuming the 30% loss of samples due to the quality, we will keep 100 samples in each group for the discovery cohorts and 150 samples in each group for the validation cohorts. Beyond the RNA-seq–based calculation, the retained cohort sizes (100 per group in the discovery cohort and 150 per group in the validation cohort, after an assumed 30% attrition for sample quality) are adequate for the proteomic, metabolomic, glycomic and machine-learning analyses. The RNASeqSampleSize estimate is conservative, as RNA-seq interrogates by far the largest feature space (~ 20,000 genes) and carries greater technical dispersion than targeted proteomic and metabolomic platforms. The Olink^®^ Reveal assay measures 1,034 proteins with high reproducibility, and the LC-MS/MS metabolomic platform quantifies ~ 1,200 features, an order of magnitude fewer tests than RNA-seq. The N-glycomic analysis similarly involves a considerably smaller number of quantified glycan features. Because these targeted platforms interrogate substantially fewer analytes with lower technical variability, the sample size derived for RNA-seq represents a conservative bound expected to provide comparable or greater statistical resolution for the proteomic, metabolomic and glycomic comparisons. For the machine-learning analyses, robustness is ensured primarily through dimensionality reduction and feature selection prior to model training, together with the prospective discovery-and-validation design and external cross-site validation.

### Data management, storage and security

The COMBAT study will generate demographic, clinical, laboratory, longitudinal outcome, and multi-omic data, including transcriptomic, proteomic, metabolomic, and glycomic datasets. These data will be integrated to support biomarker discovery, disease severity stratification, and development of AI-based predictive models.

Clinical data will be entered into a secure web-based database accessible only to authorized study personnel with two-factor authentication. All participant data will be pseudonymized before entry, and no directly identifiable personal information will be stored in the central study database. Data collected across participating centres will be harmonized and curated to support quality control and downstream analyses.

Data management procedures follow the consortium Data Management Plan and adhere to the FAIR (Findable, Accessible, Interoperable and Reusable) principles. Sensitive data will be stored on secure institutional servers at Karolinska Institutet (KI) and analysed within the National Academic Infrastructure for Supercomputing in Sweden (NAISS) (https://www.naiss.se/) using the Bianca (https://www.naiss.se/resource/bianca/) secure computing environment designed for research involving sensitive personal data. Access to study data will be limited to authorized personnel according to predefined roles and responsibilities. All the data generated in Europe will be stored in compliance with General Data Protection Regulation (GDPR) and local regulations. Indian data will be stored at Indian Biological Data Centre (IBDC), Faridabad, India.

Data sharing between consortium partners will occur through secure encrypted transfer methods and controlled-access platforms. Appropriate technical and organisational safeguards will be implemented to ensure data confidentiality, integrity, and availability throughout the study. Data processing will comply with applicable ethical, legal, and regulatory requirements, including the GDPR where applicable and relevant local regulations.

**Fig. 2:**
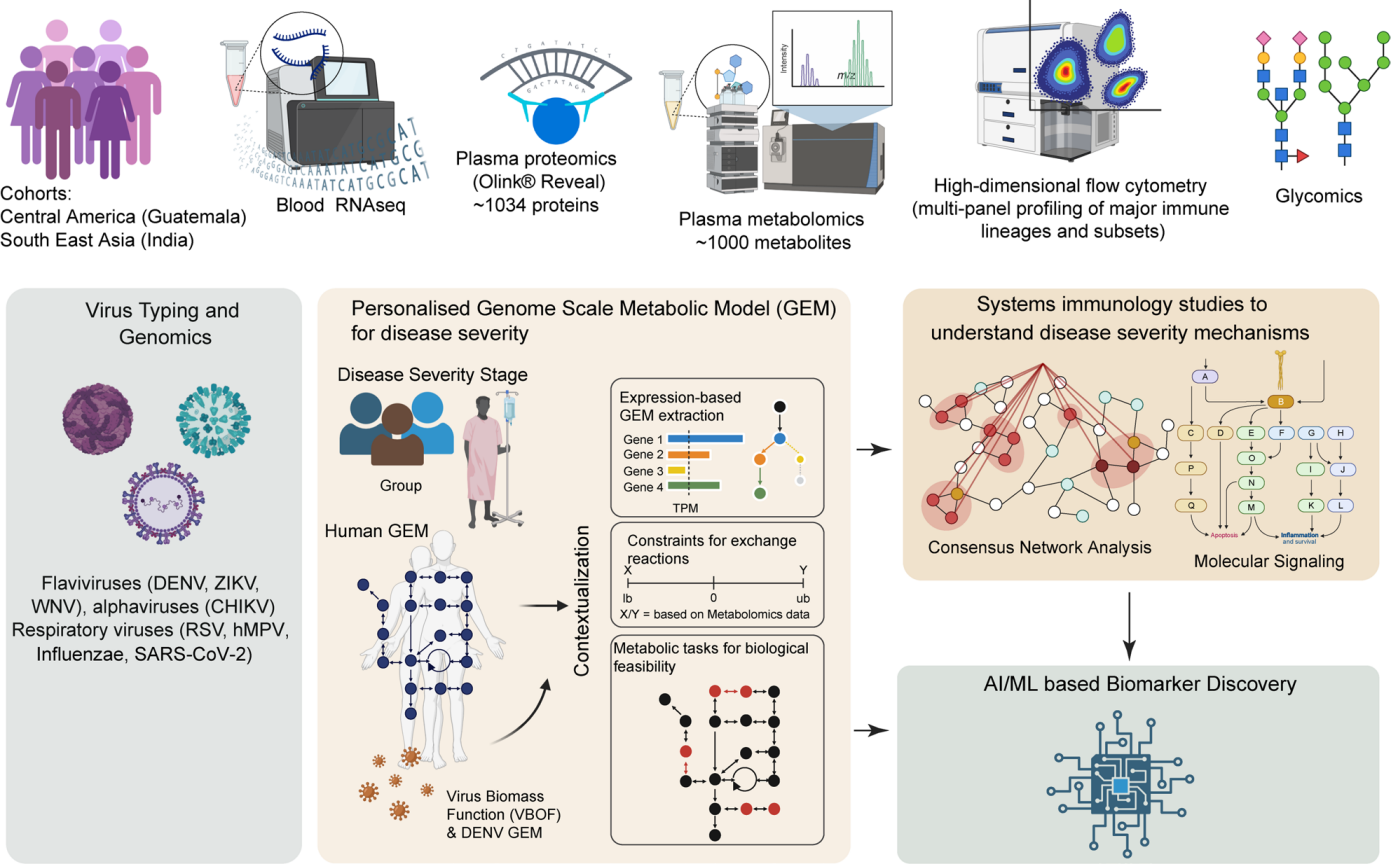
Overall study design

Following publication of the primary study results, appropriate de-identified datasets will be deposited in recognized public repositories, including the National Center for Biotechnology Information (NCBI)/Sequence Read Archive (SRA) for genomics and transcriptomics data. The mass spectrometry proteomic data will be deposited to the ProteomeXchange Consortium (http://proteomecentral.proteomexchange.org) via the PRIDE partner repository. All the codes will be available at GitHub. Overall study design is presented in Fig. 2.

### Study status

Prospective recruitment and sampling commenced in January 2025 and is ongoing at all participating sites. As of June 2026, 170 participants had been enrolled across the four study groups (DwoWS, DWS, SD and healthy controls) in the Guatemala (discovery) cohort; recruitment of the India (validation) cohort is initiated in July 2026 following the funding body approval. Recruitment is anticipated to be completed by November 2027, or earlier should the target sample size (100 participants per group in the discovery cohort and 150 per group in the validation cohort) be reached sooner. Because dengue transmission is cyclical and epidemic-dependent, recruitment timelines are contingent on local outbreak dynamics; should enrolment at any site fall behind target, pre-specified additional cohorts and biobanked samples will be accessed as a mitigation strategy, subject to approval by the COMBAT General Assembly.

### Study endpoint

#### Primary endpoints

1. Multi-omic (transcriptomic, proteomic, metabolomic and glycomic) signatures that differ significantly across dengue severity categories.
2. A validated multi-omics-derived host biomarker panel that predicts progression to severe dengue.

#### Secondary endpoints

1. Dysregulated immune and metabolic pathways associated with disease severity, identified through integrative multi-omic analysis.
2. Reproducibility of candidate biomarkers and pathways in the independent India (validation) cohort and additional external cohorts.
3. Discrimination and calibration performance of machine-learning models integrating clinical, laboratory, demographic, virological and multi-omics features for severity prediction.
4. Analytical and clinical performance of the focused 5–10 biomarker Olink^®^ Flex dengue severity panel.
5. Associations between DENV serotype, host immunometabolic profile and disease severity.

## Discussion

The COMBAT study represents one of the first large-scale, multicenter efforts designed to generate systems-level insights into host responses associated with dengue severity across diverse endemic regions. By integrating multi-omics analyses within both discovery and validation cohorts, the study aims to identify molecular signatures that could facilitate earlier recognition of patients at risk of severe disease and support improved clinical and public health decision-making. The COMBAT clinical protocol framework provides the development of mechanism-based, time-aware severity prognostic biomarkers prediction with an aim to predict the severe dengue with a consensus biomarker panel [17].

Recent epidemiological data in Guatemala illustrate the dynamic and cyclical nature of dengue transmission, as well as the inherent challenges of conducting research in endemic settings [18]. Although cumulative dengue cases in 2025 were substantially lower than during the epidemic year 2024, this decline reflects a post-epidemic phase rather than elimination of transmission. Several regions remained under alert or outbreak status, highlighting persistent vulnerability to localized resurgence. Concurrent shifts in circulating serotypes and seasonal transmission patterns further complicate the epidemiological landscape, potentially altering population immunity and influencing the risk of secondary infections and severe outcomes. These fluctuations emphasize the importance of examining host biological responses under varying transmission conditions.

Acute viral infections with pandemic potential continue to reveal critical gaps in global preparedness [17]. Their clinical outcomes vary widely, from asymptomatic infection to severe or fatal disease, creating an urgent need for biomarkers that can support early diagnosis, risk stratification, and timely intervention. Although pathogen-based tests confirm infection [19], they provide limited information on disease trajectory. Widely used prognostic markers, including IL-6, CRP, and D-dimer, provide valuable information on systemic inflammation and tissue damage but have limited disease specificity across different clinical conditions, viral etiologies, disease stages, and patient populations [20–22].

Multi-omics approaches, including proteomics, metabolomics, transcriptomics, and immune profiling, have expanded biomarker discovery by capturing integrated signatures of immune activation, metabolic rewiring, endothelial injury, and organ dysfunction [23]. In chronic and noncommunicable diseases, large-scale plasma proteomic studies have successfully identified both shared and disease-specific molecular signatures with predictive value. However, applying similar frameworks to acute viral infections is more complex because host molecular profiles change rapidly with viral kinetics, symptom onset, immune activation, treatment, and recovery. Longitudinal COVID-19 and dengue studies, show that disease severity is associated not only with specific molecular markers but also with their temporal trajectories, highlighting the importance of sampling time and disease phase [24, 25].

In dengue virus (DENV) infection, multi-omics approaches have substantially advanced our understanding of the molecular signatures linked to severe clinical outcomes, including plasma leakage, hemorrhage, and shock [26]. Transcriptomic and proteomic studies have identified early dysregulation of interferon signalling, complement activation, and platelet degranulation pathways as markers of disease severity [27–29]. In parallel, metabolomic and lipidomic analyses have revealed alterations in metabolic processes, e.g., in phospholipid metabolism, arachidonic acid signalling, and amino acid utilization, reflecting immune activation, vascular dysfunction, and endothelial injury [30, 31]. Integrative multi-omics studies further suggest that metabolic and proteomic changes may emerge before overt clinical deterioration, supporting their potential as early prognostic indicators. However, variability in sampling time, host genetic background, immune history, and co-circulating DENV serotypes continues to limit cross-study reproducibility. These challenges highlight the need for temporally harmonized, geographically stratified, and serotype-aware cohorts to enable robust biomarker validation.

Several limitations warrant consideration. As an observational study, COMBAT cannot establish causal relationships between identified molecular signatures and clinical outcomes. Differences in timing of sample collection relative to symptom onset may influence observed molecular profiles. In addition, variability in transmission patterns and diagnostic capacity across sites may affect recruitment rates and cohort composition. Although laboratory-based inclusion criteria strengthen internal validity, they may reduce the ability to capture the full clinical spectrum of dengue-like illness during low-transmission periods. Achieving an appropriate balance between methodological rigor and epidemiological representativeness remains a central challenge in cohort-based infectious disease research. Finally, although we aim to develop a unified biomarker panel applicable across both settings, differences in host genetic background, environmental exposures, and other population-specific factors may limit the identification of a fully overlapping biomarker signature. In such cases, we will develop and validate region-specific biomarker panels while also identifying conserved biological pathways that may support broader cross-population applicability.

Despite these limitations, the study has notable strengths. Its prospective, multicenter design across geographically distinct endemic regions with harmonized sample collection and protocols enables evaluation of host responses under diverse epidemiological conditions. The inclusion of both discovery and validation cohorts enhances the robustness and generalizability of findings. Moreover, the multi-omics systems biology framework provides a comprehensive platform for characterizing immune and metabolic pathways associated with disease progression. Mechanism-based biomarker discovery offers a major advantage over traditional biomarker discovery in viral diseases because it identifies markers that are directly linked to the biological processes driving disease progression [24]. Rather than relying only on statistical associations between individual markers and clinical outcomes, mechanism-based approaches integrate transcriptomic, proteomic, metabolomic, immunological, and clinical data to define the pathways underlying viral replication, immune activation, endothelial injury, metabolic dysregulation, and organ damage. This improves biological interpretability and increases the likelihood that biomarkers are reproducible across cohorts, disease stages, and populations [17].

The operational challenges encountered during implementation reflect broader weaknesses in dengue surveillance and outbreak preparedness. Dependence on clinical diagnosis, under-detection of mild cases within hospital-based systems and shifting transmission patterns contribute to uncertainty in disease burden estimates and complicate early identification of patients at risk of deterioration. By identifying host biomarkers and dysregulated pathways linked to severe outcomes, the COMBAT study seeks to advance earlier risk stratification and more informed triage decisions. Such predictive tools could support more efficient allocation of healthcare resources during outbreaks and help reduce dengue-related morbidity and mortality. Beyond its immediate relevance to dengue, the integrated clinical, laboratory, and systems biology framework developed in COMBAT offers a model for strengthening preparedness against other emerging viral infections with heterogeneous clinical manifestations. In doing so, the study advances not only dengue research but also epidemic resilience and translational public health research capacity in endemic regions.

## Data Availability

No datasets were generated or analysed during the current study.

## Declarations

### The COMBAT Consortium

Matti Sällberg^9^, Christian Giske^9^, Soham Gupta^3^, Gustaf Ahlén^9^, Jose Inzunza^9^, Mukesh Varshney^9^, Annika Charlotte Karlsson^9^, Ali Mirazimi^9^, Ákos Végvári^10^, Sofja Poznakovs^3^, Teresa Stellaccio^3^, Surender Rawat^3^, Birgitta Lindqvist^3^, Panagiotis Andriopoulos^3^, Dasja Pajkrt^11^, Adithya Sridhar^11^, Henk Marquering^11^, Nina Johannesson^11^, Amber Schotting^11^, Renata Vieira de Sá^11^, Balpreet Singh Ahluwalia^12^, Vishesh Kumar Dubey^12^, Rajkumar Savai^13^, Soni Pullamsetti^13^, Jonathan Andrew^8^, Maurizio Aiello^14^, Jacopo Furci^14^, Elisabetta Palamà^14^, Silvia Scaglione^14^, Sarantis Chlamydas^15^, Øystein Ivar Helle^16^, Goran Josipovic^17^, Irena Trbojevic-Akmacic^17^, Edson Jose Adrian Bolanos Lima^2^, Leticia Castillo Signor^2^, Kamal Singh^18^, Manjula Kalia^19^, Arup Banerjee^19^, Atul Thatai^6^ Nishi Raj Sharma^7^, Varun Surolia^7^, Priyanka Sharma^7^.

^9^Division of Clinical microbiology, Department of Laboratory Medicine, Karolinska Institutet, Stockholm, Sweden ^10^Div. of Molecular Metabolism, Dept. of Medical Biochemistry & Biophysics, Karolinska Institutet Karolinska Institutet, Stockholm, Sweden ^11^Amsterdam University Medical Center, location Academic Medical Center, University of Amsterdam, the Netherlands. ^12^University of Oslo, Norway: ^13^Justus Liebig University Giessen, Germany: ^14^React4Life, Italy ^15^Olink Ab, Sweden ^16^ChipNano Imaging Ab, Norway: ^17^Genos, Croatia ^18^University of Missouri, US ^19^Regional Center for Biotechnology, Faridabad, India.

## Abbreviations

DENV: Dengue virus
WHO: World Health Organization
DwoWS: Dengue without warning signs
DWS: Dengue with warning signs
SD: Severe dengue
ELISA: Enzyme-linked Immunosorbent Assay
AST: Aspartate Aminotransferase
ALT: Alanine Aminotransferase
Ig: Immunoglobulin
scRNA-seq: Single-cell RNA sequencing
LC-MS/MS: liquid chromatography-mass spectrometry
BBNs: Bayesian belief networks
CA: Consensus Association
GEM: Genome-scale metabolic model
AI: Artificial intelligence
SHAP: Shapley additive explanations and LIME: Local interpretable model-agnostic explanations
LIME: Local interpretable model-agnostic explanations
